# *Response to:* Abdominal CT: a radiologist-driven adjustment of the dose of iodinated contrast agent approaches a calculation per lean body weight

**DOI:** 10.1186/s41747-020-00178-x

**Published:** 2020-09-22

**Authors:** Kris A. Peet

**Affiliations:** Department of Diagnostic Radiology, QEII Health Sciences Centre, Victoria General Hospital, Dalhousie University, Victoria Building, 3rd Floor, North Wing, 1276 South Park Street, Halifax, NS B3H 2Y9 Canada

**Keywords:** Contrast media, Quality, Tomography (x-ray computed), Abdominal imaging, Liver

I read the study by Zanardo et al. entitled “Abdominal CT: a radiologist-driven adjustment of the dose of iodinated contrast agent approaches a calculation per lean body weight” [1] with interest. This study found that radiologist-derived, non-standard adjustments in contrast dosing were effective in reducing total contrast media dosing without significant impact on image quality. Our group shares a similar interest in optimizing contrast media dose, but found dissimilar results; patients randomized to a lean body weight dosing regimen in our study did not have significant reduction in the variability of mean hepatic enhancement [2]. We have several comments.

First, I agree that lean body weight may more accurately predict the volume of contrast needed; lean tissue can be expected to have a different metabolic activity and contrast uptake than adipose tissue. Previous studies in Japanese populations have demonstrated differences in enhancement per gram of contrast enhancement program of iodine [2–7] but conclusions that lean body weight dosing strategies will reduce interpatient variability have relied on inferences. A recent randomized controlled trial by our group in a North American population did not demonstrate any significant differences in contrast enhancement per gram of iodine or the variance of the mean hepatic enhancement between lean body weight dosed and total body weight dosed cohorts [2]. This is interesting, given our study design was similar on many points including the following: similar putative physiologic basis, similar measurement of lean body weight by bioelectrical impedance, as well as similar scanning protocols. Importantly, we normalized mean hepatic enhancement to iodine dose (MHE/I) in order to compare the yield of contrast media on hepatic visceral enhancement; this calculation would be very useful and interesting in the study by Zanardo et al. [1].

Second, the dosing strategy used in the study was entirely total-body-weight-based for most patients, with an *ad hoc* adjustment for doses that were subjectively considered too high. While this strategy would have the effect of decreasing the total contrast dose for patients at the high end of the range, it does not explicitly differentiate between high lean body weight and high total body weight patients, and does not account for lean patients at all.

Third, the authors state that “all CT examinations were judged as diagnostic and no patients received a repeat examination,” and have offered a proposed dose of 0.63 g of iodine per kilogram of lean body weight as a reasonable dosing standard. However, in this study, there is no formal evaluation of image quality or grading. In my experience, radiologists do not judge imaging quality enough, and the absence of such commentary within the radiologist reports does not imply diagnostic quality. In practice, the imaging quality of abdominal CT is better thought of on a sliding scale of varying quality, rather than a binary diagnostic/nondiagnostic paradigm.
